# Correlation between glycosylated hemoglobin level of patients with diabetes and cardiovascular disease

**DOI:** 10.12669/pjms.35.2.589

**Published:** 2019

**Authors:** Fengyun Wei

**Affiliations:** 1 *Fengyun Wei, CPC Shandong Prov Comm, Party Sch. 250103, China*

**Keywords:** Diabetes, HbA1c, Hypertension, Coronary heart disease

## Abstract

**Objective:**

To explore the clinical influence of the changes of glycosylated hemoglobin level of patients with diabetes on hypertension and coronary heart disease.

**Methods:**

One hundred and ninety-six patients between February 2015 and December 2016 were divided into a control group (96 non-diabetic patients) and an observation group (100 patients with diabetes) with or without diabetes. The biochemical indexes of patients in the two groups were compared. Moreover patients in the observation groups were divided into subgroups according to the presence of hypertension and coronary heart disease, and the level of HbA1c was compared between different subgroups.

**Results:**

The levels of total cholesterol (TC), triglyceride (TG) and low-density lipoprotein cholesterol (LDL-C) of patients in the two groups had no significant differences (P>0.05). However, the level of high-density lipoprotein cholesterol (HDL-C) of patients in the observation group was significantly lower than that of the control group (P<0.05). The Systolic Blood Pressure (SBP) and diastolic blood pressure (DBP), fasting plasma glucose (FPG), fasting insulin (FINS) and levels of high-sensitivity C-reactive protein (hs-CRP) and HbA1c of patients in the observation group were apparently higher than those of the control group (P<0.05). The level of HbA1c of patients with hypertension was significantly higher than that without hypertension (P<0.05). The level of HbA1c of patients with coronary heart disease was apparently higher than that without coronary heart disease (P<0.05). The Pearson correlation analysis results demonstrated that the level of HbA1c of patients in the diabetes group was in a positive correlation with SBP, DBP and level of hs-CRP (P<0.05).

**Conclusion:**

The level of HbA1c of patients with diabetes was in a positive correlation with blood pressure and level of hs-CRP. The level of HbA1c can effectively predict the occurrence of hypertension and coronary heart disease. Detecting level of glycosylated hemoglobin is of important significance in screening patients with hypertension and coronary heart disease.

## INTRODUCTION

With the constant development of living standards, the increasing incidence of chronic diseases has gradually become important.^1^ Diabetes as one of the most important chronic diseases is the most common endocrine system disease in China.^2^ Statistics have showed that the prevalence rate of diabetes is up to 9.7% among adults,^3^ its complications are numerous, and the proportion of diabetes patients with hypertension and cardiovascular and cerebrovascular diseases is increasing, which seriously threatens human health and causes severe social burden and economic burden. Therefore diabetes has attracted more and more attention.

A large number of epidemiologic studies suggested that patients with diabetes had several times higher risks of cardiovascular disease and diabetes was a continuous risk factor of cardiovascular disease.^4^,^5^ HbA1c is the product of glycation reaction of glucose and amidogen of hemoglobin under non-enzymatic catalysis, which can used as an important index reflecting glycometabolism capability. Most of patients with diabetes have abnormal HbA1c which is considered an effective index for monitoring glycicstability and therapeutic effects of medicine, which will not be affected by recent diet and medication and moreover is featured by short operation time and simple procedures.^6^ A study suggested that HbA1c was in a close correlation with cardiovascular and cerebrovascular diseases,^7^ was an independent, progressive risk factor for cardiovascular and cerebrovascular diseases, and could be used for predicting cardiovascular and cerebrovascular diseases. Khaw et al. found out the risk of cardiovascular disease of patients with diabetes increased 10 ~ 20% if HbA1c increased by 1%.^8^ In this study, the HbA1c level of patients with type 2 diabetes and its correlations with hypertension and coronary heart diseases were observed using randomize controlled trial. The level of HbA1c of patients with or without cardiovascular disease was compared. The incidence of cardiovascular disease of patients with diabetes under different levels of HbA1c was observed to explore the values of HbA1c in predicting the occurrence of cardiovascular disease of patients with diabetes. This work aims to provide a reference for the early detection and prevention of relevant chronic diseases.

## METHODS

A retrospective study was carried out on 196 patients with endocrine diseases between February 2015 and December 2016. They were divided into a control group (non-diabetes patients) and the observation group (diabetes patients) according to the presence of diabetes. The diagnosis of diabetic patients in the observation group was in accordance with the diagnostic standard of WHO for diabetes:^9^ having typical symptoms of polydipsia, polyphagia, polyuria and emaciation, 7 mmol/L higher fasting blood glucose, or 11.1 mmol/L higher random blood glucose, and 11.1 mmol/L higher two hour blood glucose with 75 g glucose tolerance test. Patients who had infectious diseases, recent hypoglycemia, cardiovascular and cerebrovascular diseases, heart and kidney dysfunction or diabetic ketoacidosis were excluded. There were 100 patients in the observation group, including 57 males and 43 females; they aged (57.68±6.33) years and had body mass index of (27.88±5.54) kg/m^2^. There were 96 patients in the control group, including 53 males and 43 females; they aged (58.13±6.96) years and body mass index of (24.12±43) kg/m^2^. There was no significant difference in gender, age and weight between the two groups. The patients signed informed consent, and the study protocol was approved by the ethics committee of our hospital.

Venous blood was collected from the patients in the fasting state in the morning. After the serum was separated, the blood lipid was detected using a Backman automatic biochemical analyzer (Beckman Coulter Inc., USA), and the indexes included total cholesterol (TC), triglyceride (TG), high density lipoprotein cholesterol (HDL-C) and low density lipoprotein cholesterol (LDL-C). The blood glucose was detected. HbA1c was measured using a 7600 automatic biochemical analyzer (Hitachi, Japan) and Kingsl glycosylated hemoglobin kit (Beijing Jiuqiang Biotechnology Co., Ltd., China) according to instructions.

### Oral glucose tolerance-insulin release test

The subjects were forbidden to take food for at least 12 hour; then elbow venous blood was extracted from each subject in the fasting state in the morning. Then each subject orally administrated the mixture of 75 h of glucose and 300 ml of water in five min; elbow venous blood was extracted after 2g. Glucose was detected using glucose oxidase method. Insulin was measured using chemiluminescent immunoassay; a fully automatic. The diagnostic criteria followed the diagnostic standard of diabetes formulated by WHO in 1999: level of fasting blood glucose higher than 7.0 mmol/L or level of random blood glucose higher than 11.1 mmol/L.

### Diagnosis of hypertension

Blood pressure was measured using a mercury sphygmomanometer in conventional procedures. The diagnostic criteria used was the diagnostic criteria mentioned in the WHO/ISH Hypertension Guidelines: SBP higher than 140 mmHg or 90 mmHg DBP.

Coronary heart disease was diagnosed using coronary arteriography according to the criterion that at least one vascular luminal stenosis exceeded 50%.

### Statistical Method

SPSS ver. 21.0 was used. Single-sample Kolmogorov-Smirnov test was used in measurement data for normality test. Normally distributed measurement data were expressed as Mean±SD, and non-normally distributed data were expressed as median. t test was used in the comparison of normally distributed measurement data between two groups; Mann-Whitney U test was used in the comparison of non-normally distributed measurement data between two groups. Count data was expressed as rate. Correlation analysis was performed using Pearson test. Difference was thought statistically significant if P<0.05.

## RESULTS

The comparison of blood lipid indexes between the two groups suggested the levels of TC, TG and LDL-C of the two groups had no significant difference (P>0.05); the level of HDL-C of the patients in the observation group was significantly lower than that of the control group (P<0.05); the SBP and DBP of patients in the observation group was obviously higher than those of the control group (P<0.05). The levels of fasting plasma glucose (FPG) and fasting insulin (FINS) of patients in the observation group were significantly higher than those in the control group (P<0.05). The levels of hs-CRP and HbA1c of patients in the observation group were significantly higher than those in the control group (P<0.05). Details are shown in Table-I.

**Table-I:** Comparison of biochemical indexes between the two groups.

Group	Observation group (n=100)	Control group (n=96)	t	P value
SBP(mmHg)	132.12±14.37	116.99±9.66	7.679	<0.05
DBP(mmHg)	81.62±10.89	75.87±9.76	6.971	<0.05
TC(mmol/L)	4.71±1.23	4.56±1.04	1.043	>0.05
TG(mmol/L)	1.06±0.30	0.81±0.24	1.127	>0.05
LDL-C(mmol/L)	2.80±0.92	2.64±0.83	1.268	>0.05
HDL-C(mmol/L)	0.82±0.30	1.11±0.37	2.453	<0.05
FPG(mmol /L)	7.97±1.12	5.13±0. 99	3.014	<0.05
FINS(mIU/L)	5.83±1.54	4.42±0.26	2.827	<0.05
hs-CRP(mg/L)	11.42±2.97	4.14±1.32	4.618	<0.05
HbA1c (%)	7.29±2.11	5.57±1. 55	3.731	<0.05

In the observation group (n=100), 63 patients had hypertension, and the incidence of hypertension was 63.0%. The level of HbA1c of patients with diabetes and hypertension was (9.38±3.11) %, while that of patients with diabetes but without hypertension was (5.97±2.01) %. Therefore the HbA1c level of the former was obviously higher than that of the latter, and the difference had statistical significance (t=6.384, P<0.05).

In the observation group, 55 patients had coronary heart disease, and the incidence of coronary heart disease was 55.0%. The level of HbA1c of patients with diabetes and coronary heart disease was (8.67±2.77) %, while that of patients with diabetes but without coronary heart disease was (5.61±1.94) %. As such the level of HbA1c of the former was significantly higher than that of the latter, and the difference had statistical significance (t=7.596, P<0.05).Pearson correlation analysis revealed that the level of HbA1c of patients with diabetes was in a positive correlation with SBP, DBP and hs-CRP (P<0.05, Table-II).

**Table-II:** Analysis on the factors associated with HbA1c of patients with diabetes.

Statistical value	SBP	DBP	hs-CRP
r value	0.781	0.786	0.714
P value	< 0.05	< 0.05	< 0.05

## DISCUSSION

HbA1c is an irreversible protein glycosylation product when glucose combines with hemoglobin in blood. Its level is related to the life of red blood cells and the average level of blood glucose in this period. It will not be affected by the transient fluctuation of blood glucose level induced by food uptake and drug use. It can reflect the average blood glucose level in the first 8~12 weeks; hence it is a suitable index for determining the degree of blood glucose control.^10^ The importance of HbA1c determination has already reached a consensus in the field of diabetes treatment, and WHO has identified HbA1c as the internationally recognized “gold standard” for diabetes monitoring.^11^ Compared with the oral glucose tolerance test, the detection of HbA1c level is simpler and has a good specificity and sensitivity in the diagnosis of diabetes. In addition to having a close relationship with diabetes, the study also showed that HbA1c was also associated with macrovascular diseases such as coronary heart disease and hypertension.^12^,^13^

The results of this study suggested that the SBP and DBP of patients with diabetes were significantly higher than that of patients without diabetes, which was consistent with the research results of Zhou.^14^ The Pearson correlation analysis pointed out that the HbA1c level of patients with diabetes was in a correlation with SBP and DBP and the level of HbA1c of patients with diabetes and hypertension was significantly higher than that of patients without hypertension, which is the innovation point of our study; content of this aspect was seldom summarized in the previous studies.^15^,^16^ The above results demonstrated that the level of HbA1c was in a positive correlation with blood pressure. There were two reasons.^17^,^18^ The first reason was that hypertension damaged endothelial function. Nitric oxide was greatly consumed by advanced glycation end products, leading to damages of nitric oxide dependent vasodilator, weakening of angiotasis and increase of blood pressure. The second reason was that insulin accelerated reabsorption of water and sodium in distal renal tubule, which induced increase of blood volume, and insulin excited sympathetic nervous system, which promoted peripheral vasoconstriction and increase of blood pressure through acting on catecholamine.

Some studies have showed that HbA1c is one of the important reasons inducing cardiovascular disease.^19^ Cardiovascular disease, especially coronary heart disease, is mainly caused by atherosclerosis caused by hypercholesterolemia and hyperlipidemia. It has been pointed out that high HbA1c contribute to atherosclerosis.^20^ HbA1c reflects that the control level of blood glucose of patients. High level of HbA1c indicates that patients are at a state of high blood sugar for a long time. NO and prostacyclin produced by human endothelial cells can inhibit thrombosis though inhibiting platelet aggregation. In the state of hyperglycemia, the direct toxicity of glucose reduces the replication of endothelial cells, weakens the repair capacity, and damages endothelial cell; as a result, the release of endothelin increases, and the release of NO and prostacyclin decreases, damaging vasomotoricity and weakening the inhibition on thrombogenesis.^21^ Moreover HbA1c can weaken the deformation capacity of erythrocyte through increasing the viscosity of erythrocyte and reducing the fluidity and moreover slows the dissociation rate of oxygenated hemoglobin, which is an important factor of tissue hypoxia.^22^ Therefore, the rise of HbA1c is likely to increase the risk of atherosclerosis. Farrag AAM et al. found that insulin resistance was common among patients with diabetes and moreover the content of HbA1c increased, which could directly affect myocardial cell energy metabolism, aggravate hemodynamic disorder and induce coronary heart disease.^23^ The results of this study showed that the HbA1c level in the diabetic patients with coronary heart disease was significantly higher than that in the non-coronary heart disease group. The Pearson correlation analysis indicated that the HbA1c level of the diabetic patients was related to the level of hs-CRP, which accords with the analysis of the above mechanism and similar to the findings of Ametov et al.^24^

### Limitations of the study

The sample size was small and the samples all came from single center. We intend to carry out prospective multi-center studies with large sample size to further explore the correlations between glycated hemoglobin level, hypertension and coronary heart disease.

## CONCLUSION

The level of HbA1c of patients with diabetes is in a positive correlation with the level of hs-CRP. Detection of HbA1c level can effectively predict the occurrence of hypertension and coronary heart disease. The monitoring of HbA1c level should be strengthened in clinics. Diabetic patients with high level of HbA1c should pay attention to the prevention and screening of hypertension and coronary heart disease.
